# Marine-Inspired Ovothiol Analogs Inhibit Membrane-Bound Gamma-Glutamyl-Transpeptidase and Modulate Reactive Oxygen Species and Glutathione Levels in Human Leukemic Cells

**DOI:** 10.3390/md23080308

**Published:** 2025-07-30

**Authors:** Annalisa Zuccarotto, Maria Russo, Annamaria Di Giacomo, Alessandra Casale, Aleksandra Mitrić, Serena Leone, Gian Luigi Russo, Immacolata Castellano

**Affiliations:** 1 Department of Molecular Medicine and Medical Biotechnology, University of Naples Federico II, 80131 Naples, Italy; annalisa.zuccarotto@unina.it (A.Z.); ales.casale@studenti.unina.it (A.C.); 2 National Research Council, Institute of Food Sciences, 83100 Avellino, Italy; maria.russo@isa.cnr.it (M.R.); annamariadigiacomo90@gmail.com (A.D.G.); gianluigi.russo@cnr.it (G.L.R.); 3 Institute of Clinical and Molecular Virology, Friedrich-Alexander University Erlangen-Nürnberg, 91054 Erlangen, Germany; aleksandra.mitric27@gmail.com; 4 Department of Biology and Evolution of Marine Organisms, Stazione Zoologica Anton Dohrn, Villa Comunale, 80121 Naples, Italy; serena.leone@szn.it

**Keywords:** ovothiols, 5-thiohistidine, gamma-glutamyl-transpeptidase, glutathione, GGT inhibitors, leukemic cells

## Abstract

The enzyme γ-glutamyl transpeptidase (GGT), located on the surface of cellular membranes, hydrolyzes extracellular glutathione (GSH) to guarantee the recycling of cysteine and maintain intracellular redox homeostasis. High expression levels of GGT on tumor cells are associated with increased cell proliferation and resistance against chemotherapy. Therefore, GGT inhibitors have potential as adjuvants in treating GGT-positive tumors; however, most have been abandoned during clinical trials due to toxicity. Recent studies indicate marine-derived ovothiols as more potent non-competitive GGT inhibitors, inducing a mixed cell-death phenotype of apoptosis and autophagy in GGT-overexpressing cell lines, such as the chronic B leukemic cell HG-3, while displaying no toxicity towards non-proliferative cells. In this work, we characterize the activity of two synthetic ovothiol analogs, L-5-sulfanylhistidine and iso-ovothiol A, in GGT-positive cells, such as HG-3 and HL-60 cells derived from acute promyelocytic leukemia. The two compounds inhibit the activity of membrane-bound GGT, without altering cell vitality nor inducing cytotoxic autophagy in HG-3 cells. We provide evidence that a portion of L-5-sulfanylhistidine enters HG-3 cells and acts as a redox regulator, contributing to the increase in intracellular GSH. On the other hand, ovothiol A, which is mostly sequestered by external membrane-bound GGT, induces intracellular ROS increase and the consequent autophagic pathways. These findings provide the basis for developing ovothiol derivatives as adjuvants in treating GGT-positive tumors’ chemoresistance.

## 1. Introduction

Glutamyl transpeptidase (GGT) is an enzyme localized on the cellular membrane, where it catalyzes the hydrolysis of extracellular glutathione (GSH, γ-L-glutamyl-L-cysteinylglycine) [1]. It is synthesized as a unique polypeptide, which undergoes an autocleavage into a large and a small subunit, interacting to form a functional dimer with the active site directed towards the extracellular space [2]. Human GGTs are glycoproteins, and their molecular mass varies, due to the different degrees of protein glycosylation [3,4]. The physiological substrate GSH is biosynthesized in the cytosol and is considered the most abundant antioxidant thiol in the cell [5]. Under oxidative stress conditions, GSH can be oxidized to its disulfide GSSG, and the excess of both forms can be effluxed into the extracellular environment through multidrug-resistance-associated proteins [6,7]. There, membrane-bound GGT cleaves the peculiar γ-glutamyl bond between glutamate and the amino group of cysteine in GSH [8,9]. Then, membrane-bound dipeptidases hydrolyze the resulting cysteinyl–glycine to release glycine and cysteine, which are finally transported into the cell by neutral amino acid transporters [10]. In the cell, these amino acids serve as a source for the de novo synthesis of GSH and proteins [11]. Therefore, in humans, where GSH acts as a key antioxidant molecule, GGT plays a pivotal role in GSH metabolism and in balancing cellular redox homeostasis [12,13,14]. Previous work reported that GGT-deficient mice display delayed growth, associated with significantly higher GSH concentrations in plasma (175 μM) and urine (15.4 μM) compared to wild-type mice (27.6 μM and 6.2 μM, respectively), and ultimately die due to cysteine deficiency [15]. These mice are more susceptible to oxidative stress and lung injury than wild-type mice [16]. Several human tumors, including hepatocellular carcinoma and renal cell carcinoma, exhibit high levels of GGT activity, which enhances their ability to recycle GSH [17,18,19,20]. Consequent elevated intracellular GSH levels contribute to chemo- and radiotherapy resistance and prevent the initiation of the apoptotic cascade in tumor cells [20]. When inhibiting GGT activity for 2 h, the intracellular cysteine concentration in GGT-positive tumors was significantly reduced [21,22]. In leukemia cells, the drug-resistant lines displayed 3-fold higher GGT activity and intracellular GSH levels, allowing cells to metabolize more extracellular GSH and take up more cysteine [23]. Several studies have suggested that the same mechanism could confer GGT-positive tumors resistance to pro-oxidant anticancer therapies, including platinum-based compounds, alkylating agents, and radiation [19,23]. Therefore, intense efforts have been continuously devoted to identifying new GGT inhibitors to potentially ameliorate the treatment of pathologies associated with high levels of GGT activity, such as cancer, ischemia–reperfusion-induced renal injury, asthma, and liver fibrosis [24].

The first compounds to show GGT-inhibitory activity included glutamine analogs and other aminoacidic derivatives such as acivicin, 6-diazo-5-oxo-L-norleucine, azaserine [25,26], sulfur derivatives of L-glutamic acid, and γ-(monophenyl) phosphonoglutamate analogs [27]. However, most of these compounds have displayed toxicity due to their interference with essential metabolic pathways [24,25,26]. Among monophenyl phosphonoglutamate analogs, only the butanoic acid derivative GGsTop has been reported to be less toxic [27] and effective for treating asthma and oral mucositis [28,29]. Another class of less toxic and uncompetitive inhibitors has been developed from benzene-sulfonamide (OU749) [30]. Recently, OU749 has been encapsulated in a supramolecular platinum prodrug nano-assembly delivery system for the chemotherapy of cisplatin-resistant cancer [31]. This strategy has been shown to efficiently reduce GGT activity and intracellular GSH levels, thereby augmenting peroxides and preventing chemoresistance. We have recently focused on a class of 5-(*N*)-methyl thiohistidines, sulfur-containing natural products, known as ovothiols [32,33], which play a key role in controlling the cellular redox balance thanks to their ability to perform redox exchange with GSH [24,32]. Natural ovothiol A (ovo), isolated and characterized from the eggs of the sea urchin *Paracentrotus lividus* and abundant in other marine invertebrates [34], has been reported to inhibit membrane-bound GGT when administered in its disulfide form, and concomitantly induce autophagy in a human liver carcinoma cell line HepG2 and chronic B leukemic cells HG-3 [35,36,37]. Moreover, ovo has been reported to exhibit GGT-inhibitory activity and an anti-inflammatory action on the livers of mice affected by liver fibrosis [38]. Overall, these studies have prompted the chemical synthesis and testing of marine-inspired ovo analogs [39].

Here, we report the biological effect of two synthetic ovo-related compounds—2-amino-3-(5-sulfanylimidazol-4-yl) propanoic acid, here referred to as L-5-sulfanyl histidine (5-thio), and 2-amino-3-(1-methyl-5-sulfanylimidazol-4-yl) propanoic acid, here referred to as iso-ovothiol A (iso-ovoA)—on GGT activity and GSH metabolism in human leukemic cells. Our results suggest that these compounds could be potentially employed as adjuvants for treating GGT-positive tumors and other diseases associated with elevated GGT activation.

## 2. Results

### 2.1. Marine-Inspired Ovothiol Analogs Inhibit Membrane-Bound GGT in GGT-Positive Cell Lines

**Figure 1:**
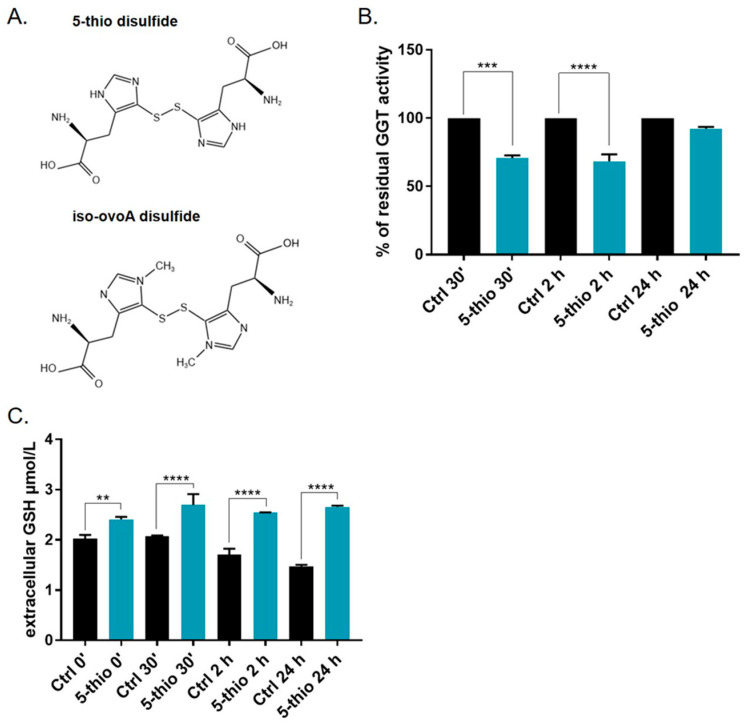
Disulfide structures of 5-thio and iso-ovoA (**A**). Membrane-bound GGT activity determination at different times after 30 µM 5-thio treatment in HG-3 (**B**). The percentages of GGT residual activity were compared to 100% activity of not-treated cells (Ctrl). Extracellular GSH determination via HPLC (**C**). The amounts of GSH at different times of 30 µM 5-thio treatment in HG-3 cells were compared to that in non-treated cells. A one-way ANOVA was used with a Tukey post hoc statistical analysis. Symbols indicate significance: ** *p* < 0.01; *** *p* < 0.001; **** *p* < 0.0001.

HepG2 and HG-3 cells were previously tested for GGT expression and activity and recognized as a rich source of GGT [36]. Partially purified GGT extracts from HepG2 and HG-3, containing the mature membrane-bound form of GGT, were tested for residual GGT activity in the presence of 5-thio and iso-ovoA at different concentrations. The first compound, 5-thio, represents the unmethylated precursor of natural ovo. The second, iso-ovoA, differs from the natural compound due to the methylation site on the imidazole ring of histidine (Figure 1A). The two synthetic compounds were used in the disulfide form to avoid side reactions, as their thiol group displays a low pKa, associated with extreme reactivity [32]. Under these conditions, 50% GGT inhibition in HepG2 extracts was obtained at ~36 µM for 5-thio and at ~59 µM for iso-ovoA. Similarly, in HG-3 extracts, 50% GGT inhibition was obtained at ~32 µM for 5-thio and at ~54 µM for iso-ovoA. These results indicate that 5-thio induces significant inhibition of GGT, and is comparatively more efficient than iso-ovoA. Moreover, HepG2 extracts appeared slightly more resistant to GGT inhibition, likely due to the high grade of protein glycosylation [36]. Therefore, we continued evaluating GGT’s residual activity on HG-3 cells, using 5-thio as the stronger ovothiol analog inhibitor. To confirm that 5-thio inhibits membrane-bound GGT in dividing cells, HG-3 cells were treated with 30 µM of 5-thio, and residual GGT activity was measured after 30 min, 2 h, and 24 h. GGT-membrane-bound activity was significantly reduced after 30 min and 2 h of treatment in the presence of 5-thio (Figure 1B). On the contrary, we did not observe a significant reduction in GGT activity at 24 h. The inhibition of the membrane-bound GGT activity was confirmed by the increase in GSH levels in the extracellular medium at different incubation times, measured via an HPLC analysis (Figure 1C).

### 2.2. Ovothiol Analogs Are Not Cytotoxic in Human Leukemia Cells

**Figure 2:**
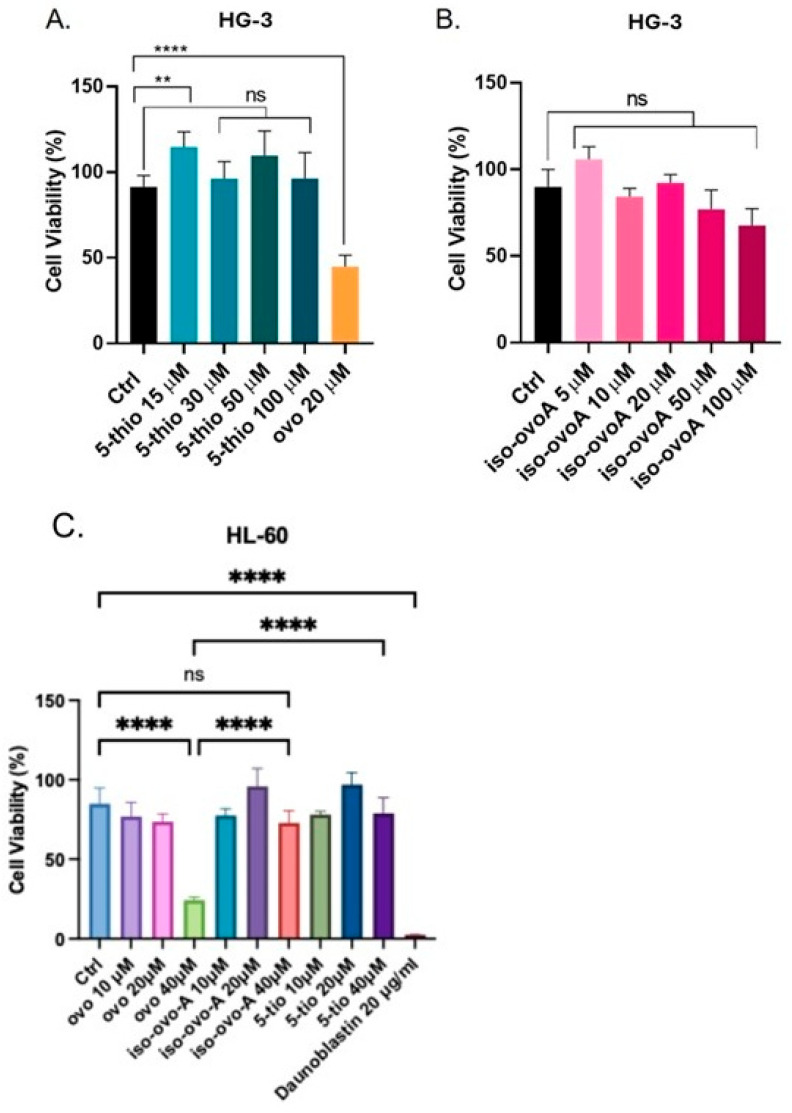
The HG-3 cells were treated with specified concentrations of the indicated compounds for 24 h at 37 °C in a 5% CO_2_ incubator using a complete RPMI medium. The Cy-Quant cell viability assay was employed to evaluate the cytotoxicity of the reported concentrations of natural ovo and its analogs, 5-thio (**A**) and iso-ovoA (**B**). HL-60 cells were incubated with the indicated compounds, and a cell viability assay (Cy-Quant) was performed after 24 h (**C**). Daunoblastin, a cytotoxic drug, was used as a positive control. The fluorescence from viable cells (excitation at 485 nm/emission at 530 nm) was expressed as a percentage of untreated (Ctrl) cells. The bars in the graphs represent the mean of two independent experiments, each performed in quadruplicate. A one-way ANOVA was used with a Tukey post hoc statistical analysis. Symbols indicate significance: ** *p* < 0.01; **** *p* < 0.0001; ns: not significant.

To investigate the effects of marine-inspired ovo derivatives on cell proliferation, HG-3 and HL-60 cells of acute promyelocytic leukemia, both known for their GGT expression [36,40], were exposed to 5-thio and iso-ovoA at various concentrations for 24 h, and the results were compared with those of natural marine ovo. Treatment of HG3 with 5-thio showed no decrease in cell viability from 15 µM to 100 µM, with a slight increase noted at 15 µM (Figure 2A). Similarly, treatment of HG-3 cells with iso-ovoA at concentrations up to 100 µM did not lead to a significant decrease in cell viability, as measured by the CyQuant assay for proliferating cells (Figure 2B). In contrast, natural ovo caused a significant reduction in HG3 cell viability at a concentration of 20 µM (Figure 2A). Similarly, the treatment with 5-thio and iso-ovoA did not significantly alter the cell viability of HL-60 cells (Figure 2C). On the contrary, natural ovo induced a significant reduction in HL-60 cell viability at 40 µM (Figure 2C).

### 2.3. 5-Thio Does Not Modify Autophagy Flux in GGT-Positive HG-3 Cell Line

**Figure 3:**
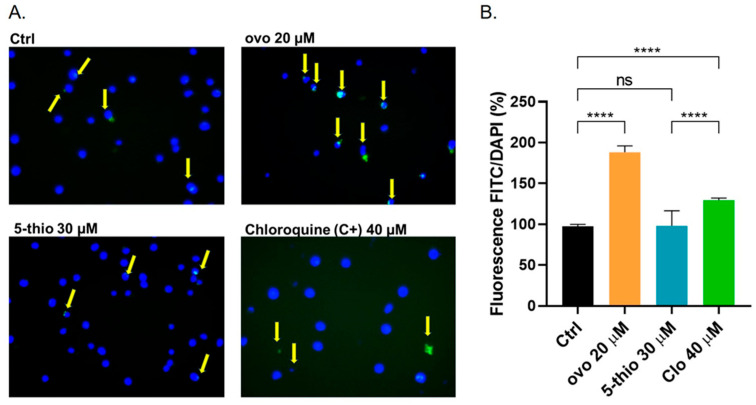
The HG-3 cells were treated with indicated concentrations of different compounds for 24 h at 37 °C with 5% CO_2_ in a complete RPMI medium. Cyto-ID dye and Hoechst nuclear staining were added to the cells and incubated for 30 min. Cytoplasmic autophagosomes were monitored with an inverted microscope, and representative images were taken as reported in (**A**). Specific autophagosome staining (yellow arrows indicate the FITC-positive cells), calculated as fluorescence from the FITC/DAPI ratio, is expressed as a percentage of untreated (Ctrl) cells (**B**). Chloroquine (40 μM) was used as a positive control. In (**B**), bars indicate the mean of two experiments in quadruplicate. A one-way ANOVA was used with a Tukey post hoc statistical analysis. Symbols indicate significance: **** *p* < 0.0001; ns: not significant.

We previously demonstrated that natural ovo induces cytotoxic autophagy in HepG2 and HG-3 cells [35,36]. Here, we investigated whether a similar effect could be observed with the chemical derivative 5-thio, which inhibits human GGT with a slightly lower but significant strength. We quantified autophagic vacuoles using a Cyto-ID autophagy kit, which incorporates a green dye that fluoresces when taken up by pre-autophagosomes, autophagosomes, and autolysosomes. The green fluorescence, normalized to the total number of cells stained with Hoechst nuclear dye, revealed an increased presence of autophagic vacuoles in cells treated with ovo at 20 µM compared to untreated control cells (Figure 3A,B), indicating altered autophagic flux. In contrast, treatment with 5-thio at 30 µM did not lead to significant changes relative to untreated cells. As a positive control, HG-3 cells were treated with 40 µM of chloroquine for 24 h (Figure 3A,B), which by blocking autophagy flux caused an accumulation of cytoplasmic autophagosomes. Under these conditions, fluorescence microscopy and image capture (micrographs in Figure 3A) confirmed the activation of cytotoxic autophagy by ovo in HG-3 cells.

### 2.4. Bioavailability of 5-Thio in GGT-Positive HG-3 Cell Line

**Figure 4:**
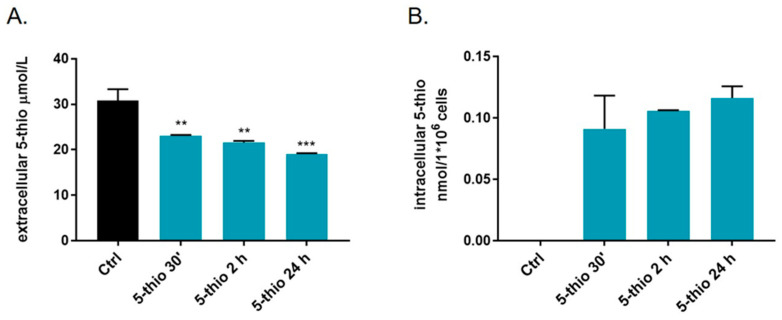
Determination of 5-thio bioavailability in the cell medium of HG-3 cell lines following drug treatment (**A**). Ctrl represents positive control, i.e., culture medium added with 30 µM 5-thio. Intracellular levels of 5-thio detected via HPLC at different times of 30 µM 5-thio treatment in HG-3 (**B**). Ctrl represents negative control (not treated cells, which have not incorporated 5-thio). A one-way ANOVA was used with a Tukey post hoc statistical analysis. Symbols indicate significance: ** *p* < 0.01; *** *p* < 0.001.

To assess the bioavailability of 5-thio in GGT-positive cell lines, we detected the extracellular and intracellular levels of 5-thio upon treatment of HG-3 cells at different times via HPLC, after derivatization of the samples with 4-bromomethyl-7-methoxycoumarin (BMC). We observed a significant time-dependent decrease in the extracellular levels of 5-thio at 30 min up to 24 h (Figure 4A). However, our data indicated that most of the administered 5-thio remained outside the cells, compatible with its interaction with GGT. On the other hand, we detected a significant peak of 5-thio in the intracellular extract of HG-3 cells treated with 5-thio soon after treatment, from 30 min to 24 h (Figure 4B). On the contrary, we did not observe any peak corresponding to natural ovo inside the cells after treatment of GGT-positive cell lines such as HepG2 [35].

### 2.5. 5-Thio Modulates Intracellular ROS and GSH Levels in GGT-Positive HG-3 Cell Line

**Figure 5:**
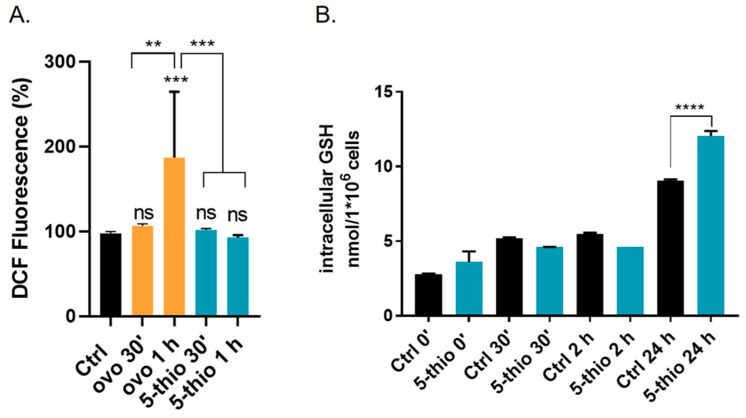
(**A**) Intracellular peroxide levels measured with fluorescent probe DCF in HG-3 cells treated for 30 min or 1 h with natural ovo or 5-thio at the indicated concentration. Peroxide levels were expressed as a percentage of DCF fluorescence (excitation at 485 nm/emission at 530 nm) compared to untreated cells (Ctrl). Data represent the mean of two experiments in quadruplicate. (**B**) Determination of intracellular GSH levels in HG-3 cell lines after 30 µM 5-thio treatment, compared to untreated cells (Ctrl). A one-way ANOVA was used with a Tukey post hoc statistical analysis. Symbols indicate significance: ** *p* < 0.01; *** *p* < 0.001, **** *p* < 0.0001; ns: not significant.

To investigate whether 5-thio, when administered in its disulfide form, can undergo redox exchange with GSH in the reducing intracellular environment, we assessed the levels of intracellular ROS using the CM-DCF-DA probe. As shown in Figure 5A, treatment with 30 µM of 5-thio resulted in a slight but not significant alteration of intracellular ROS levels after 30 min (>5%), while 20 µM ovo induced a higher increase (>10%) at the same time point. However, after 1 h of treatment with 30 µM of 5-thio, intracellular peroxide levels remained unchanged (Figure 5A). In contrast, treatment with 20 µM of natural ovo led to a significant increase in intracellular ROS after 1 h (>50%). On the other hand, the levels of intracellular GSH, detected via HPLC, significantly increased at 24 h of treatment with 5-thio compared to untreated cells (Figure 5B), likely suggesting an initial redox exchange of 5-thio with intracellular GSH, followed by an increase in GSH biosynthesis.

## 3. Discussion

Due to its involvement in GSH metabolism, drug detoxification, and the maintenance of cellular redox homeostasis, GGT plays a key role in the progression of different types of cancer [24,38]. In particular, in human tumors, where GGT is overexpressed, it induces new protein synthesis, cell proliferation, and resistance to chemo- and radiotherapy [23,41,42,43,44]. Therefore, some therapeutic strategies involve using GGT inhibitors to sensitize GGT-overexpressing tumors to chemotherapy [19,23]. For example, the administration of the GGT inhibitor acivicin in combination with chemotherapy in mice resulted in reduced intracellular GSH levels and the regression of metastatic melanoma in most of the animals tested [22,42]. Unfortunately, most GGT inhibitors developed to date, including acivicin, are too toxic for humans or do not display the efficacy desired for clinical use [24,25]. Therefore, recent research has been devoted to discovering novel potent and nontoxic GGT inhibitors. Recently, we have demonstrated that the marine natural ovo strongly inhibits GGT activity and induces a mix of apoptosis and autophagy in GGT-overexpressing cell lines, including HepG2 and HG-3 [35,36]. However, the purification of such a compound from natural sources, such as sea urchins, requires many efforts and a large number of eggs [35]. This does not represent an ecologically sustainable task, also considering that sea urchins are a protected species. Therefore, in this work, we have investigated the GGT-inhibitory potential of two ovo analogs obtained via biomimetic chemical synthesis [39]. We previously confirmed that HepG2 and HG-3 cells overexpress GGT compared to nonmalignant tissue [36], making them a suitable target for testing potential GGT inhibitors. Using GGT activity assays, we observed that both compounds can inhibit GGT extracted from both HepG2 and HG-3 cells. In particular, 5-thio achieves a 50% reduction in enzymatic activity at a concentration of ~30 µM in HG-3 cells, which are more sensitive to GGT inhibition compared to HepG2, likely due to the lowest degree of GGT glycosylation. On the other hand, iso-ovo A is less potent than 5-thio, and both compounds display lower potency compared to natural ovo (*Ki* = 21 µM) [36]. Our findings appear particularly surprising if we compare the results of cytotoxicity assays. Indeed, 5-thio and iso-ovo A did not display any significant effect on the cell vitality of leukemic cells, such as HG-3 and HL-60, both overexpressing GGT [36,40]. On the other hand, natural ovo caused significantly reduced cell vitality at 20 µM in HG-3 and 40 µM in HL-60 cells, confirming the ability to induce cell death through an autophagic pathway [36].

**Figure 6:**
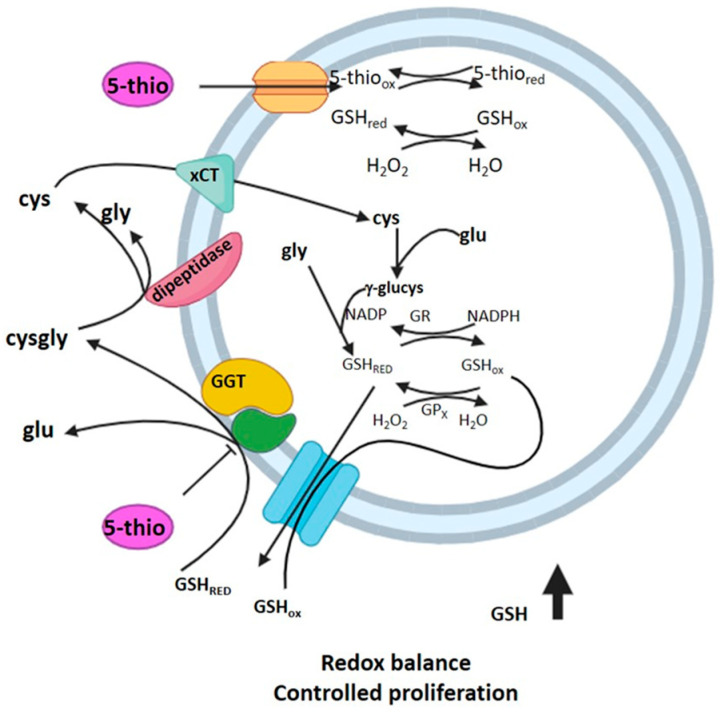
Proposed model of 5-thio action on HG-3 cells. Upon administration of 5-thio disulfide to HG-3 cells, a portion of the compound interacts with membrane-bound GGT, inhibiting its activity and subsequently increasing extracellular GSH levels. Meanwhile, the remaining free 5-thio may enter HG-3 cells through a not yet identified membrane transporter. Inside the cell, 5-thio disulfide is reduced by the intracellular pool of reduced GSH, thereby contributing to ROS scavenging and maintaining redox homeostasis.

By inhibiting GGT activity and the extracellular GSH hydrolysis, we expect to find an increase in the extracellular pool of GSH and a decrease in the intracellular GSH levels [22,42]. This is the case for natural ovo, which was previously observed to inhibit membrane-bound GGT in HepG2 and HG-3 and induce the accumulation of extracellular GSH [36]. Therefore, to verify the occurrence of a similar behavior for the chemical derivative 5-thio, we employed thiol derivatization coupled with an HPLC analysis to measure the levels of GSH inside and outside HG-3 cells upon treatment with 5-thio. Indeed, we observed the accumulation of extracellular GSH in the medium, following 5-thio-induced inhibition of membrane-bound GGT activity (Figure 1). Furthermore, we observed the uptake of intracellular 5-thio with its concomitant decrease in the extracellular medium (Figure 4). This indicates that part of the free 5-thio, not involved in the interaction with membrane-bound GGT, entered the cells, where the intracellular pool of GSH is expected to reduce it [39,45]. Once reduced, intracellular 5-thio may act as an antioxidant and contribute to the fight against the increased pool of ROS in cells (Figure 6). This is likely why we observed only a slightly but not significant increased level of intracellular ROS after 30 min of 5-thio treatment, followed by a balance of intracellular ROS at 1 h of treatment. On the contrary, ovo supplementation increased intracellular ROS levels at 30 min and more significantly after 1 h of treatment (Figure 5A). Indeed, since ovo is a more potent inhibitor of GGT compared to 5-thio, the effects of its action on the decrease in intracellular GSH levels should be more evident compared to 5-thio. In our previous work, we did not detect ovo inside GGT-positive cells such as HepG2, thereby suggesting that most ovo in its disulfide form interacts with membrane-bound GGT and the free form does not enter the cells at appreciable levels [35]. This may explain why, in the case of ovo, the effect on the inhibition of GGT and the blockage of GSH recycling overcomes the potential of the thiol to act as an intracellular antioxidant. Indeed, in the case of ovo, we observed an increase in intracellular ROS, which likely contributes to the induction of a mixed phenotype of autophagy and apoptosis [36]. This phenotype was not observed upon treatment with the synthetic analog 5-thio, which in fact did not significantly inhibit HG-3 cell proliferation compared to natural ovo. In this case, we observed an increase in intracellular GSH compared to untreated cells at 24 h, which enables tumor cells to maintain a favorable redox balance, thereby possibly contributing to escaping cell death pathways, following 5-thio treatment (Figure 6).

The different biological actions of natural ovo compared to 5-thio and iso-ovoA may be ascribed to slight differences in their chemical properties. Indeed, the disulfide bond of natural ovo is stronger (115.24 kcal/mol) and more resistant to reduction than those of 5-thio (111.94 kcal/mol) and iso-ovoA (99.4 kcal/mol) [46]. The strength and length of the disulfide bridge can modulate redox exchange with GSH differently, thereby resulting in a different biological effect. Indeed, 5-thio may be more easily reduced by intracellular GSH than natural ovo, thereby acting as an antioxidant. Moreover, it is worth noting that the inhibition of purified equine GGT by ovo and 5-thio was observed only with the disulfide form of the compounds [36,37]. Therefore, the lower GGT inhibition power of 5-thio and iso-ovoA compared to natural ovo may be correlated to their lower resistance to reduction. Our previous docking analysis predicted that disulfide forms of ovo and 5-thio interact with key residues in the donor and the acceptor binding sites of human GGT; on the contrary, the reduced forms interact only with residues in the donor binding site, thereby explaining the stronger inhibitory activity of both molecules in disulfide compared to the reduced ones [37]. Furthermore, the small predicted distance between the –OH group of the catalytic Thr in GGT and the disulfide bond of ovo (2.62 Å) and 5-thio (2.36 Å) suggested a mechanism of GGT inhibition involving the initial binding of the disulfide form of these molecules, with the formation of an ovoS–enzyme complex and the consequent release of the reduced forms. This may explain the recovery of GGT enzymatic activity following pre-incubation with ovo and 5-thio compared to inactivated irreversible enzyme complexes [36], and why we did not observe significant membrane-bound GGT inhibition after 24 h of 5-thio treatment in HG-3 cells (Figure 1B).

The finding that 5-thio inhibits human GGT and does not affect cell vitality or autophagic flux in HG-3 could provide an incentive for its potential use as an adjuvant in treating GGT-positive tumors’ chemoresistance or ameliorating other pathologies characterized by high levels of GGT activity. On the other hand, the dual action of natural ovo as a reversible and non-competitive GGT inhibitor and inducer of death pathways in GGT-positive cells may point to its potential role as an anti-tumoral drug. Future experiments will be devoted to understanding whether chemical derivatives of 5-thio, used in combination with chemotherapeutic drugs, can prevent GGT-positive tumors’ chemoresistance or ameliorate pathologies associated with high activity levels of GGT.

## 4. Materials and Methods

L-5-sulfanylhistidine and iso-ovothiol A were kindly provided by Jean Claude Yadan, Tetrahedron Company (Paris, France) and prepared as described in [39]. The ^1^H-NMR and ^13^C-NMR spectra of L-5-sulfanylhistidine derivatives are reported in the Supplementary Materials of [39]. Ovothiol A was purified from sea urchin *P. lividus* eggs, as previously described in [35]; its purity was checked via an LC-MS analysis and ^1^H-NMR and ^13^C-NMR data are reported in [35].

### 4.1. Enzyme Isolation and GGT Activity Assay

GGT-enriched extracts were prepared from HepG2 and HG-3 cell lines as described by Brancaccio et al. with some modifications [36]. The Hep-G2 cell line, derived from a human hepatocellular carcinoma, was generously provided by Professor M.A. Belisario of Salerno University. The HG-3 lymphoblastoid cell line was obtained by Professor A. Rosen from the Department of Clinical and Experimental Medicine, Division of Cell Biology, Linköping University, Sweden. Cells were homogenized by a potter in 4 volumes of 25 mM Tris-Cl at pH 7.5, containing 0.33 M of sucrose, 0.2 mM of EDTA, and Halt Protease and Phosphatase Inhibitor Cocktail (Merck/Sigma-Aldrich, Milan, Italy). Then re-suspended cells were centrifuged at 9000× *g* for 20 min and the supernatant was spun at 24,000× *g* for 1 h. The new pellet, resuspended in 25 mM of Tris-Cl at pH 7.35, 0.5% Triton X-100, and Phosphatase Inhibitor Cocktail, was centrifuged again at 24,000× *g* for 1 h. A colorimetric test was used to assay the supernatant for GGT activity. The assay buffer contained 100 mM of Tris-Cl at pH 8; each reaction contained 1 mM of GpNA as a donor substrate and 20 mM of GlyGly as an acceptor substrate. The formation of the product, p-nitroaniline, was continuously monitored at room temperature at 412 nm using a Cary 100 UV–Vis spectrophotometer (Agilent Technologies, Milan, Italy). The residual activity was reported as a percentage of GGT activity in the absence of the compounds. All substrates for GGT assay (GpNA, GlyGly) were purchased from Merck or Sigma-Aldrich (Burlington, MA, USA).

### 4.2. Derivatization and HPLC Analyses

Cell pellets (5 × 10^6^ cells) were disrupted in 50 µL of 1X PBS and centrifuged for 20 min at 13,000 rpm and 4 °C. Cytosolic extracts and 1 mL of culture medium were lyophilized overnight and rehydrated with 20 µL of MQ water. Subsequently, each sample was derivatized following the protocol of Russo et al. [47]. Briefly, 100 µL of extraction buffer (acetonitrile: HClO_4_ 0.75 M, 1:2) was added to the rehydrated samples and vortexed for 30 s. Extracts were centrifuged for 10 min at 16,000 rpm, and 15 µL of 2 M K_2_CO_3_ was added to 100 µL of cleared lysates. Insoluble cell debris and excesses of K_2_CO_3_ were removed via another 10 min centrifugation phase at 16,000 rpm. Then, 100 µL of supernatants was basified with 10 µL of 50 mM Li_2_CO_3_ and then reduced with 3 µL of 200 mM DTT followed by a 5 min incubation phase at room temperature. Then, 25 µL of BMC 20 mM in dimethyl sulfoxide (DMSO) was added and the samples were incubated for 30 min in the dark. Finally, the samples were acidified with 10 µL of 10% formic acid, vortexed for 30 s to remove CO_2_, and centrifuged to remove excess BMC. The samples (20 μL) were examined via reversed-phase HPLC utilizing an Agilent Infinity 1260 apparatus (Agilent Technologies, Santa Clara, CA, USA) equipped with a Poroshell 120-C 18 column (4 μm, 150 × 4.6 mm, Agilent). Detection of thiol-BMC conjugates was accomplished by measuring absorbance at 330 nm. The column was equilibrated with a mixture of 98% solvent A (0.1% trifluoroacetic acid in water) and 2% solvent B (0.1% trifluoroacetic acid in acetonitrile) at a flow rate of 0.8 mL/min. The gradient used in [48] was modified as follows: 0.0–2 min, 2% B; 2.0 min, 3% B; 2–6 min, 3–9% B; 6–21 min, 9–45% B; 21–23 min, 45–90% B; 23–26 min, 90% B; 26–27 min, 90–2% B; 27–37, 2% B.

The thiol–BMC conjugates’ identification was allowed by measuring absorbance at 330 nm. The presence of intra- and extracellular 5-thio and GSH in the samples was verified via coelution with standards of these two compounds. The quantification of both metabolites was obtained by integrating the areas of the corresponding peaks in the HPLC chromatogram, using a calibration curve built with standards of 5-thio and GSH.

### 4.3. Cell Culture and Viability Assays

HL-60 cells (acquired from the European Collection of Cell Cultures (ECACC) and distributed in Italy by Merck-Sigma (Milan)) [49] and HG-3 cells [50] were cultured in RPMI medium supplemented with 10% FBS, 1% L-glutamine, and 1% penicillin/streptomycin (EuroClone, Milan, Italy) at 37 °C in a humidified atmosphere with 5% CO_2_. The concentrations of various molecules used in each experiment are specified in the corresponding figure legends. HG-3 and HL-60 cells were treated with designated concentrations of these molecules for 24 h at 37 °C in complete RPMI medium. Next, Cy-Quant dye and its background suppressor reagent (Invitrogen, Life Technologies, Milan, Italy) were added, and cells were incubated for 1 h at 37 °C in 5% CO_2_ [51]. The fluorescence from viable cells (excitation at 485 nm/emission at 530 nm) is displayed in the graphs as a percentage of untreated (Ctrl) cells using a Synergy HT multiwell reader (Synergy HT BioTek, Milan, Italy).

### 4.4. Intracellular ROS Measurement

Cells were incubated at a concentration of 2 × 10^4^ cells/mL for 30 min with a peroxide-selective probe, the chloromethyl derivative of di-chloro-fluorescein diacetate (CM-DCFDA), at a concentration of 10 µM (Invitrogen, Life Technologies, Milan, Italy). The incubation was performed in phosphate-buffered saline (PBS) at 37 °C in a humidified 5% CO_2_ atmosphere. Fluorescence was measured using a Synergy HT reader (BioTek, Milan, Italy) with an excitation wavelength of 485 nm and an emission wavelength of 530 nm [52].

### 4.5. Autophagy Assay

The Cyto-ID autophagy detection kit (Enzo Life Sciences, Milan, Italy) was used to assess autophagy status following incubation with different compounds, as described in the figure legends. This kit employs a cationic amphiphilic tracer that allows precise quantification of autophagosomes within cells. The green dye, excitable at 488 nm, is formulated to minimize lysosome staining and shows bright fluorescence when incorporated into pre-autophagosomes, autophagosomes, and autolysosomes. After incubation, cells were washed and a mixture of the autophagy detection marker (Cyto-ID) and nuclear dye (Hoechst 33342) was added. Autophagosome numbers were quantified by comparing green fluorescence (Cyto-ID) to blue fluorescence (Hoechst) using a microplate fluorescence reader (Synergy HT). Microphotographs were captured with a fluorescence-inverted microscope (Axiovert Zeiss, Milan, Italy) at 400× magnification, using either DAPI or fluorescein isothiocyanate (FITC) filters after staining [53].

### 4.6. Statistical Analysis

Statistical analyses of data were performed via a one-way ANOVA, and differences among different treatments were evaluated using Tukey’s post hoc test (*p* < 0.05). The analyses were performed using the software package Prism 6 (GraphPad Software Inc., San Diego, CA, USA).

## 5. Conclusions

For the first time, we provide evidence of significant differences in the biological actions of the natural product ovo and its synthetic derivatives. Natural ovo not only acts as a potent GGT inhibitor but also as an inducer of cytotoxic autophagy in GGT-positive cells; in contrast, the chemical derivatives are less effective as GGT inhibitors and do not compromise cell viability or autophagic flux in these cells. This biological divergence appears to stem from slightly different chemical properties, which result in different mechanisms of action. Natural ovo primarily binds and inhibits external membrane-bound GGT, increasing extracellular GSH and intracellular ROS levels, thereby triggering apoptotic and autophagic pathways (see highlighted image). Conversely, 5-thio, displaying weaker GGT-inhibitory activity, interacts partially with external GGT while entering HG-3 cells, where it likely acts as an antioxidant through redox exchange with GSH. Consequently, while natural ovo shows potential as an anti-tumoral drug, the chemical derivatives may serve as adjuvants in treating GGT-positive tumors’ chemoresistance or in mitigating other pathologies, characterized by high levels of GGT activity. A novel concept that emerged in this work is the dual role of natural ovo as an antioxidant or pro-oxidant, depending on the target and cellular context. In non-proliferative cells that do not overexpress GGT, natural ovo partially enters the intracellular environment [45], where it acts as an antioxidant or anti-inflammatory compound [45,54]. However, in proliferating, GGT-positive cells, its primary action as a GGT inhibitor results in pro-oxidant effects. This duality underscores the complex and context-dependent therapeutic potential of ovo and its derivatives.

## Data Availability

The datasets supporting this article will be made available on request.

## Abbreviations

The following abbreviations are used in this manuscript:

ANOVA	Analysis of Variance
BMC	4-Bromomethyl-7-Methoxycoumarin
CO_2_	Carbon Dioxide
Ctrl	Control
DAPI	4′,6-Diamidino-2-Phenylindole
DMSO	Dimethyl Sulfoxide
DTT	Dithiothreitol
FBS	Fetal Bovine Serum
FITC	Fluorescein Isothiocyanate
GGT	Gamma-Glutamyl Transpeptidase
GSH	Glutathione
GSSG	Glutathione Disulfide
HPLC	High-Performance Liquid Chromatography
iso-ovoA	Iso-Ovothiol A
LC-MS	Liquid Chromatography–Mass Spectrometry
NMR	Nuclear Magnetic Resonance
ns	Not Significant
ovo	Ovothiol A
OU749	GGT Inhibitor OU749
PBS	Phosphate-Buffered Saline
RPMI	Roswell Park Memorial Institute (Medium)
ROS	Reactive Oxygen Species
TFA	Trifluoroacetic Acid
5-thio	L-5-Sulfanylhistidine
